# Dosimetric characteristics of a reusable 3D radiochromic dosimetry material

**DOI:** 10.1371/journal.pone.0180970

**Published:** 2017-07-13

**Authors:** Jong Min Park, So-Yeon Park, Chang Heon Choi, Minsoo Chun, Ji Hye Han, Jin Dong Cho, Jung-in Kim

**Affiliations:** 1 Department of Radiation Oncology, Seoul National University Hospital, Seoul, Korea; 2 Institute of Radiation Medicine, Seoul National University Medical Research Center, Seoul, Korea; 3 Biomedical Research Institute, Seoul National University Hospital, Seoul, Korea; 4 Center for Convergence Research on Robotics, Advance Institutes of Convergence Technology, Suwon, Korea; Mizoram University, INDIA

## Abstract

**Purpose:**

To investigate the dosimetric characteristics of PRESAGE^REU^ dosimeters.

**Methods:**

Commercially available PRESAGE^REU^ dosimeters (size of 10 mm × 10 mm × 45 mm) were divided into two groups, with one of the groups placed at room temperature of 22°C (RT group) and another group placed at low temperature of 10°C (LT group). A total of 3 dosimeters (set of dosimeters) were irradiated at a time, with doses of 1 Gy, 2 Gy, 4 Gy, 8 Gy, 12 Gy, 16 Gy, and 20 Gy, at a nominal dose rate of 400 MU/min at temperature of 22°C. The dosimeters were irradiated three additional times by delivering the same doses as those during the initial irradiations (4 irradiation cycles). Optical density (OD) was assessed using optical CT scanning.

**Results:**

Considering both linearity and sensitivity of the OD curves, R^2^ above 0.95 and sensitivity above 0.04 ΔOD/Gy were observed at the 1^st^ irradiation (reading time ≤ 6 h) and 2^nd^ irradiation (reading time = 0.5 h) for the RT group. For the LT group, those values were observed at the 1^st^ irradiation (reading time ≤ 2 h), and the 3^rd^ and 4^th^ irradiations (both reading times = 0.5 h). Considering the reproducibility of signals in response to the same dose, dosimeters in the RT group showed average deviations among dosimeters less than 5% (the 1^st^ and 2^nd^ irradiations at the reading time of 0.5 h), while for dosimeters in the LT group showed average deviations among dosimeters less than 6% (the 3^rd^ and 4^th^ irradiations at the reading time of 0.5 h). For the rest, the OD curves were not linear, sensitivities of the dosimeters were lower than 0.04 ΔOD/Gy, and OD deviations at the same dose were larger than 6%.

**Conclusions:**

At room temperature, PRESAGE^REU^ dosimeters could be used for dose measurement only for up to two dose measurement sessions. At low temperatures, usage of PRESAGE^REU^ dosimeters for dose measurement seems to be possible from the 3^rd^ irradiation. When reusing PRESAGE^REU^ dosimeters, the OD curve should be re-defined for every measurement session because the shape of this curve depends on the irradiation history.

## Introduction

Intensity modulated radiation therapy (IMRT) and volumetric modulated arc therapy (VMAT) can deliver prescription doses to target volumes while minimizing doses to radiosensitive organs at risk (OARs) located near the target volumes [1, 2]. By virtue of this capability, IMRT and VMAT can achieve superior tumor control while at the same time reducing radiotherapy-associated complications. To apply IMRT and VMAT in the clinical setting, careful verification of a treatment plan by measuring the delivered dose distribution is essential before proceeding to treating patients, because the inverse planning procedures of IMRT and VMAT are not intuitive [3, 4]. In addition, IMRT and VMAT are more susceptible to errors because those techniques generally generate steep dose gradients near the target volumes [3, 4]. In this respect, pre-treatment patient-specific quality assurance (QA) for both IMRT and VMAT is highly recommended and routinely performed in the clinical setting [3, 4]. Pre-treatment QA typically involves the measurement of a planar dose map of some kind, followed by two-dimensional (2D) gamma evaluation [5].

Recently, several studies questioned the clinical relevance of 2D gamma passing rates [6–8]. Nelms *et al*. showed that 2D gamma passing rates are not correlated with clinically relevant dose-volumetric parameters for IMRT with intentionally introduced delivery errors [7]. Park *et al*. also demonstrated that 2D gamma evaluation likely does not provide sufficient information for detecting small delivery errors in VMAT [8]. Kim *et al*. demonstrated that no correlations were observed between the 2D and quasi three-dimensional (3D) gamma passing rates, for both IMRT and VMAT [6]. Therefore, there is a need to develop novel verification methods for both IMRT and VMAT in the clinical setting, for generating more information on the delivered dose distributions, such as those in the 3D gamma evaluation.

To measure 3D dose distributions directly, various 3D gels have been developed and tested [9–18]. Adamovics and Maryanski developed the PRESAGE^TM^ dosimeter (Heuris Inc., Skillman, NJ), which is a clear polyurethane plastic doped with leuco dyes, *i*.*e*., radiochromic components [9]. When a PRESAGE dosimeter is irradiated, radiolytic oxidation of leuco dyes takes place, inducing a color change [9]. The color change of the PRESAGE dosimeter following its exposure to ionizing radiation can be quantified by measuring changes in the optical density (OD) using optical computed tomography (CT) [10]. Several studies reported the dosimetric characteristics of PRESAGE dosimeters [12, 19, 20]. Although PRESAGE dosimeters have some advantages, such as rigidity, machinability, and robustness to the environment (except UV irradiation), one important disadvantage is that this dosimeter is disposable. To overcome this disadvantage, PRESAGE^REU®^ (Heuris Inc., Skillman, NJ) was developed and recently introduced in the clinical setting [14]. Similar to PRESAGE, PRESAGE^REU^ changes its color when exposed to ionizing radiation. However, the color change after irradiation is fading at room temperature with the PRESAGE^REU^ dosimeter eventually relaxing to its original state. When the PRESAGE^REU^ dosimeter returns to its original state, it can be reused for measuring another dose distribution, *i*.*e*., multiple uses of the PRESAGE^REU^ system are in principle possible. Given this attractive characteristics, it is surprising that only a few studies addressed this property of PRESAGE^REU^. Pierquet *et al*. investigated the dosimetric characteristics of PRESAGE^REU^ [14]. These authors demonstrated reusability of PRESAGE^REU^ by performing several repeated irradiations. Cho *et al*. developed a software tool that compared the calculated 3D dose distribution to the one measured using PRESAGE^REU^, and the results were used for pre-treatment patient-specific QA [11]. In this study we investigated the dosimetric characteristics of PRESAGE^REU^. We investigated the behavior of PRESAGE^REU^ on multiple re-irradiations, by analyzing the linearity and sensitivity of OD curves and by analyzing the dose-rate dependency and temperature dependency of this dosimeter.

## Materials and methods

### Irradiation of PRESAGE^REU^

**Fig 1:**
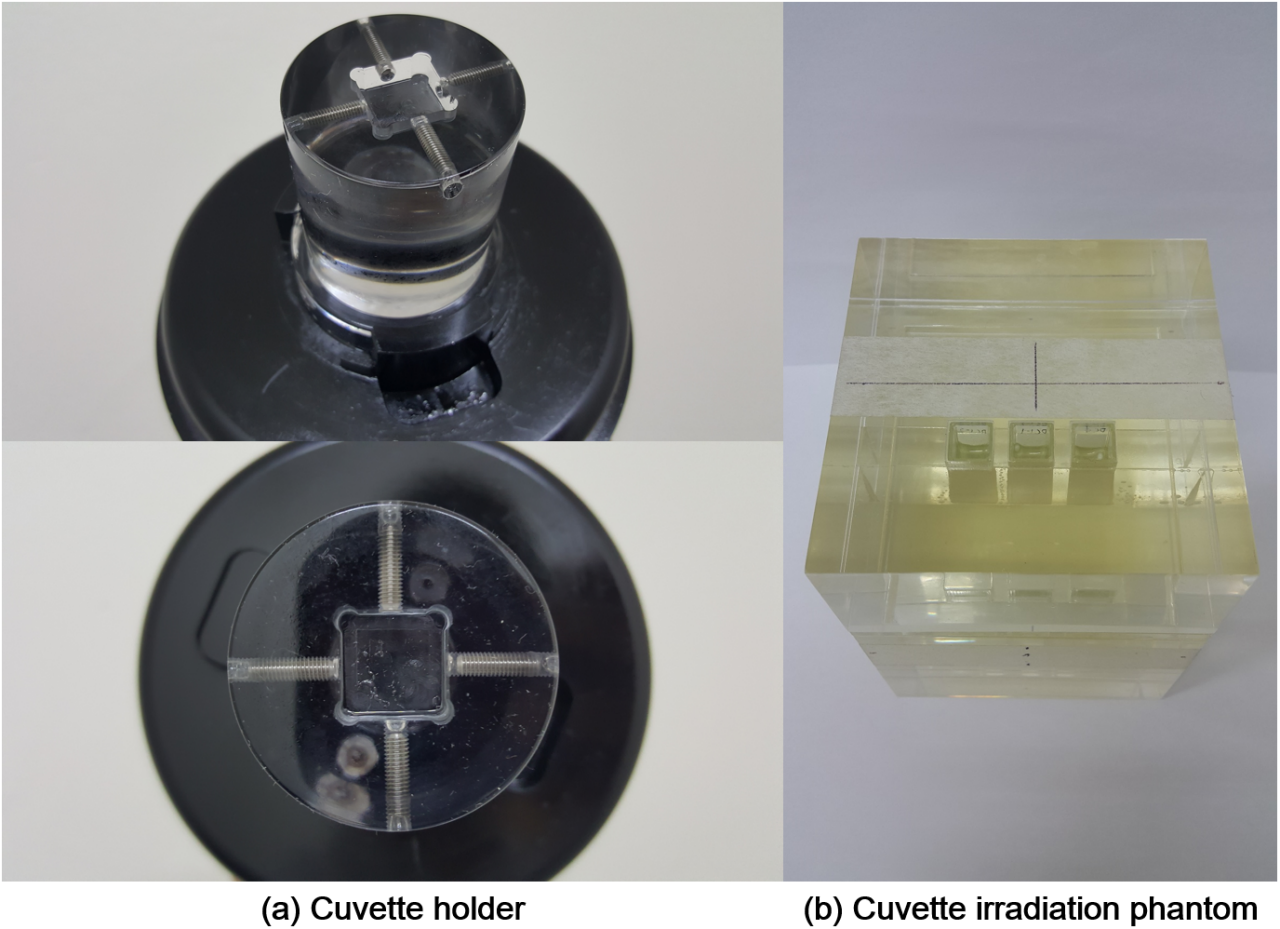
**A custom-made cuvette holder (a) and a custom-made mold phantom for irradiation of a total of 3 cuvettes (b)**. For a reproducible fixation of cuvettes during scanning with optical CT, we made a custom-made holder with acrylic body. In the holder, there were four screws for fixing a cuvette. For irradiating the three cuvettes by given uniform doses, a mold phantom made of acrylic was fabricated. The dimensions of the phantom were 10 cm × 10 cm × 14 cm, and the cuvettes were located at the center, with inter-cuvette separation of 0.5 cm. The centers of the cuvettes were located at the depth of 7 cm into the phantom.

We investigate the dosimetric characteristics of commercially available PRESAGE^REU^ that was poured into a standard spectrometer cuvette with the dimensions of 10 mm × 10 mm × 45 mm (Heuris Inc., Skillman, NJ). During the whole period of experiment, all the cuvettes were kept in a light shielding case in order to avoid UV exposure since the UV radiation is known to be efficient in causing a color change of the PRESAGE^REU^ [21]. A custom-made mold phantom, made of acrylic, was fabricated to insert cuvettes for delivering a uniform dose to a total of 3 cuvettes at a time. The phantom dimensions were 10 cm × 10 cm × 14 cm. Three cuvettes could be located at the center of the phantom, *i*.*e*., the center of each cuvette was located at the depth of 7 cm into the phantom. In addition, we designed a cuvette holder as another custom-made device for reproducible fixation of the cuvettes during scanning with optical CT (Vista^TM^ Optical CT Scanner, Modus Medical Devices Inc., Ontario, Canada). These custom-made devices are shown in Fig 1.

**Fig 2:**
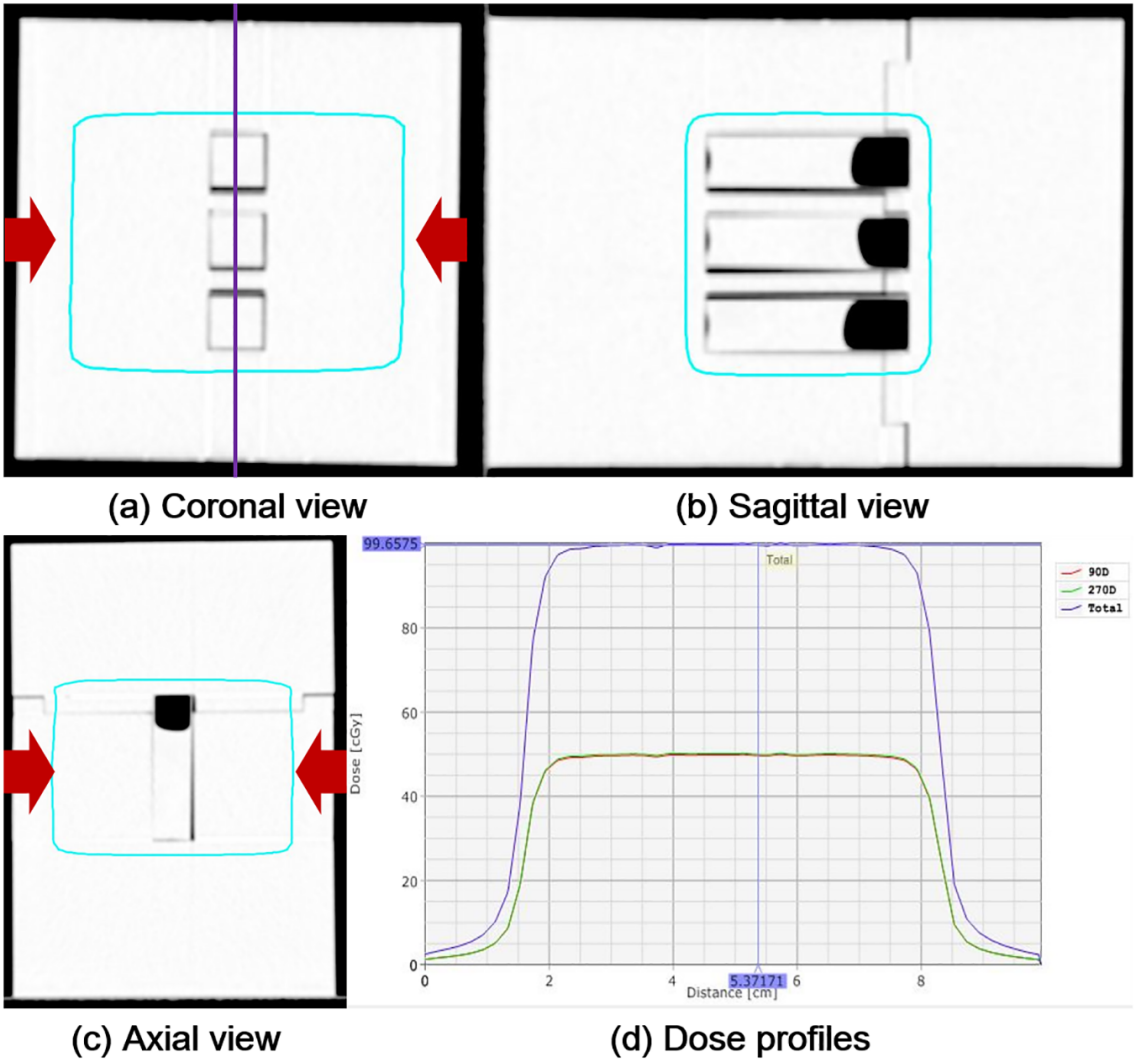
**The prescribed isodoses in the coronal (a), sagittal (b), and axial (c) views, as well as the dose profiles along the sagittal direction.** The prescribed isodose lines are shown in the cyan color. The beam directions were shown in red arrows. The dose profiles in the phantom along the sagittal direction of a field with the gantry angles of 270° and 90° are shown in the green and red colors, while the summed dose profile is shown in the violet color. The line where the dose profiles were acquired is shown in violet color in the coronal view.

Three cuvettes were inserted into the mold phantom, and CT images were acquired using a Brilliance CT Big Bore^TM^ (Phillips, Cleveland, OH), with the imaging slice thickness of 1 mm. Using these CT images, IMRT plans using two opposed bilateral beams, *i*.*e*., two fields with gantry angles of 90° and 270°, were generated to deliver uniform doses to all of the cuvettes at a time. The IMRT plans were generated with 10 MV photon beams in the flattening filter-free (FFF) mode of TrueBeam^TM^ STx (Varian Medical Systems, Palo Alto, CA) in the Eclipse^TM^ system (Varian Medical Systems, Palo Alto, CA). The isocenter was located at the centroid of the mold phantom. A dose volume optimizer (DVO, ver.10, Varian Medical Systems, Palo Alto, CA) was used for optimizing the IMRT plan, and the anisotropic analytic algorithm (AAA, ver.10, Varian Medical Systems, Palo Alto, CA) was used for calculating the dose, with the calculation grid size of 1 mm. The isodose lines intended to be delivered to all of the cuvettes inside the mold phantom, and the dose profiles across the cuvettes, are shown in Fig 2. The maximum and minimum doses in the isodose lines were 99.8% and 100.5% of the prescription dose, respectively. We optimized once to generate an IMRT plan and we multiplied that IMRT plan varying the prescription doses from 1 Gy to 20 Gy in order to deliver various doses to the PRESAGE^REU^.

To investigate the effect of temperature on PRESAGE^REU^, we divided the PRESAGE^REU^ cuvettes into two groups. The cuvettes in the first group were always at room temperature, which was ~22°C (the room temperature group, the RT group). On the other hand, the cuvettes in the second group were in a refrigerator that maintained the temperature at 10°C (the low temperature group, the LT group). Temperature consistency was determined every 12 h for the PRESAGE^REU^ cuvettes in both the RT and LT groups. Before the initial irradiation of the PRESAGE^REU^ cuvettes, the cuvettes in the LT group were kept in the refrigerator for two days. During irradiation, the PRESAGE^REU^ cuvettes were at room temperature in the treatment room. After the initial irradiation, the PRESAGE^REU^ cuvettes were returned to the refrigerator. For scanning the PRESAGE^REU^ cuvettes in the LT group with optical CT to determine OD changes (ΔOD) induced by irradiation, the cuvettes were taken out from the refrigerator to the room temperature environment. Immediately after scanning, the cuvettes were returned to the refrigerator. After completing several scans with optical CT post irradiation, every cuvette in this study was maintained at room temperature for 7 days to ensure color relaxation before starting the next irradiation. Then, the cuvettes of the LT group were kept in the refrigerator for 2 days before the next irradiation. Before each irradiation and for each PRESAGE^REU^ dosimeter we checked the stability of OD. If the OD was stable, we proceeded to scan the PRESAGE^REU^ dosimeter to obtain the background level for the next irradiation. Therefore, the cuvettes in the LT group were at room temperature only when irradiating, scanning with optical CT, and 7 days of relaxation before the next irradiation.

For every irradiation, to examine the consistency of readings of the PRESAGE^REU^ dosimeters on the same doses, a set of three cuvettes were irradiated at a time, as shown in Fig 2. Various doses were delivered to the sets of cuvettes in both groups; these doses were 1 Gy, 2 Gy, 4 Gy, 8 Gy, 12 Gy, 16 Gy, and 20 Gy, delivered at a nominal dose rate of 400 MU/min. We investigated the dosimetric characteristics of PRESAGE^REU^ for doses up to 20 Gy, because such a large dose could be delivered at a single fraction in stereotactic ablative radiotherapy (SABR). For every irradiation, a set of non-irradiated cuvettes were as a control group for eliminating environmental effects.

To examine the dose-rate dependency, we additionally delivered doses of 1 Gy, 8 Gy, and 20 Gy, at a dose rate of 2400 MU/min, in the FFF mode, to the different sets of cuvettes. The behavior of PRESAGE^REU^ irradiated at a high dose rate (nominal dose rate of 2400 MU/min, 351.1 ± 4.3 cGy/min) was compared to that of PRESAGE^REU^ irradiated at a low dose rate (nominal dose rate of 400 MU/min, 109.0 ± 0.2 cGy/min).

To investigate the reusability of PRESAGE^REU^, we re-irradiated the cuvettes additional three times by delivering the same doses as those delivered in the initial irradiations. Therefore, each PRESAGE^REU^ cuvette was irradiated four times, *i*.*e*., each was subject to 4 cycles of irradiation, and multiple scans were performed for both the RT and LT groups.

### Reading of PRESAGE^REU^

After irradiation, OD changes were acquired using an optical CT scanner at 0.5 h, 2 h, 4 h, 6 h, 12 h, 24 h, 48 h, and 84 h following irradiation (8 scans for a single irradiation session). The optical CT scanner is designed to work with radiochromic dosimeters which have an absorption peak at 590 nm or 633 nm and we used a red light of a 633 nm wavelength for scanning. Before irradiation, all PRESAGE^REU^ dosimeters were scanned to acquire background 3D OD distributions, *i*.*e*., background information. After the 3D OD distributions for the irradiated cuvettes were reconstructed, the previously acquired background 3D OD distributions were subtracted from the reconstructed 3D OD distributions to eliminate background signals and imaging artefacts. To match the index of refraction of PRESAGE^REU^ in the aquarium of optical CT, we prepared a solution by mixing octyl salicylate with octyl methoxycinnamate, as recommended by the manufacturer (octyl salicylate:octyl methoxycinnamate = 0.908:0.092), and filled it into the aquarium during scanning. For scanning and 3D reconstruction of the project images, VistaScan^TM^ and Vista 3-D Reconstruction^TM^ programs (Modus Medical Devices Inc., Ontario, Canada) were used, respectively. The camera frame rate, the number of projections per scan, the projection angle increment, the image voxel size, the camera resolution and the camera shutter speed were 3.75 fps, 512, 0.703125°, 0.5 mm, 640 × 480, and 0.025 s, respectively. The OD data for the reconstructed 3D images were acquired using the MicroView^TM^ software (Parallax innovations, Ontario, Canada). The region of interest (ROI) at the center of a cuvette was defined as a 2 mm × 2 mm × 2 mm cube, and for each cuvette, we reported ODs averaged over all voxels in the ROI.

## Results

### The time-dependent decay of PRESAGE^REU^ signals

**Fig 3:**
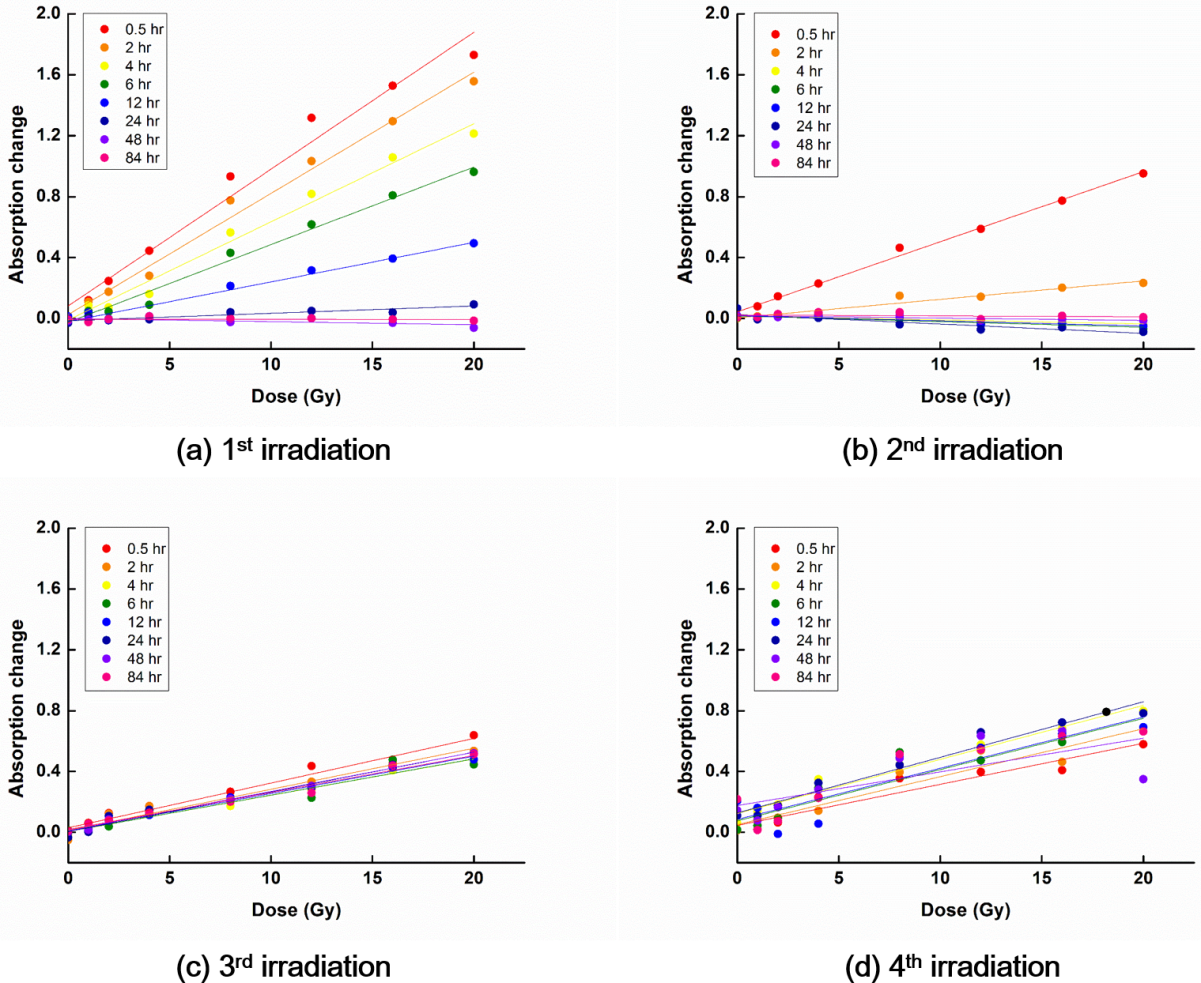
OD curves for dosimeters at room temperature. In general, the slope of the OD curve decreased with increasing the reading time after irradiation. Although the slope of the OD curve in general decreased with increasing PRESAGE^REU^ reuses, the trend was not very pronounced.

**Fig 4:**
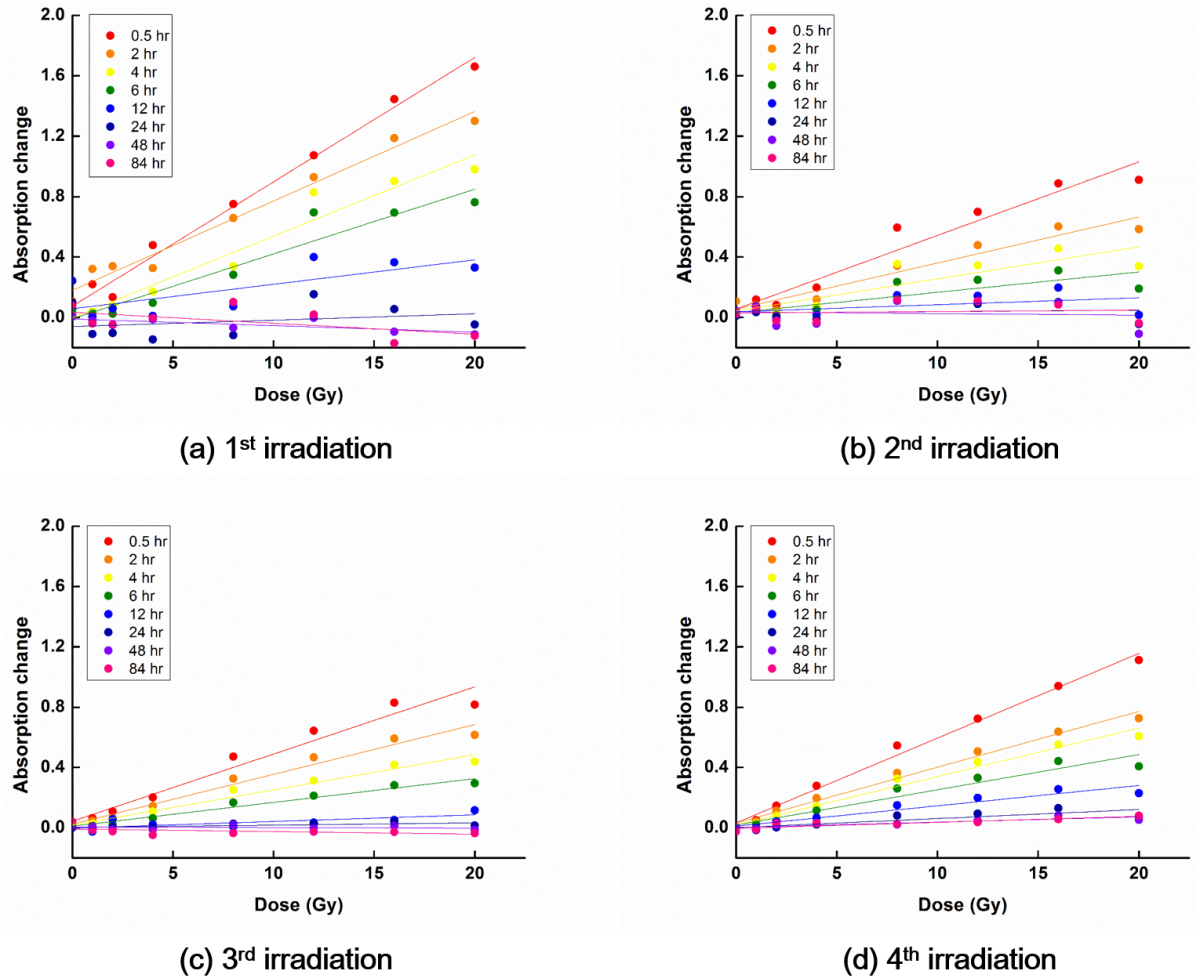
OD curves for dosimeters at low temperature. In general, the slope of the OD curve decreased with increasing the reading time after irradiation. The slope of the OD curve after the initial irradiation was steeper than those after re-irradiations.

The temporal decay characteristics of PRESAGE^REU^ signals following irradiation are shown in Figs 3 and 4 for the RT and LT groups, respectively.

For the RT group, the signal related to the 1^st^ irradiation decayed to the background level in 48 h following the irradiation. The signals related to the 2^nd^ irradiation decayed more rapidly than those related to the 1^st^ irradiation, reaching the background level in 6 h following the irradiation. In the cases of the 3^rd^ and the 4^th^ irradiations, no distinctive decay tendencies were observed and the signals related to these irradiations were not fully cleared out.

For the LT group, the signals related to the irradiations decayed to the background level in 48 h following the irradiations, regardless of the irradiation history. It appears that the decay aspects stabilized for the 3^rd^ and 4^th^ irradiations. Most of the remaining signals generally cleared out with the course of time.

### Linearity of the OD curves

The R^2^ values of the OD curves for the RT and LT groups are summarized in Table 1. The linearity of the OD curves generally became worse as the reading time after irradiation (reading time) of PRESAGE^REU^ increased, for both RT and LT groups.

**Table 1:** The values of R^2^ for the linear fits to the OD curves.

**Reading time after irradiation (hours)**	1^st^ irradiation	2^nd^ irradiation	3^rd^ irradiation	4^th^ irradiation
	Room temperature			
0.5	0.973	0.994	0.963	0.904
2	0.988	0.942	0.958	0.910
4	0.984	0.520	0.982	0.966
6	0.983	0.827	0.928	0.875
12	0.959	0.771	0.987	0.789
24	0.799	0.778	0.968	0.954
48	0.355	0.493	0.992	0.459
84	-0.163	-0.093	0.979	0.825
	Low temperature			
0.5	0.987	0.938	0.955	0.992
2	0.974	0.922	0.971	0.985
4	0.943	0.740	0.969	0.978
6	0.913	0.636	0.963	0.928
12	0.446	0.063	0.508	0.907
24	-0.078	-0.150	0.418	0.667
48	0.480	-0.157	-0.109	0.704
84	0.231	-0.151	0.095	0.763

For dosimeters in the RT group, R^2^ was above 0.9 for the reading time less than 2 h. Among the OD curves for dosimeters in the RT group, the OD curve obtained at 0.5 h after the 2^nd^ irradiation was the most linear (R^2^ = 0.994).

For dosimeters in the LT group, R^2^ was above 0.92 for the reading time less than 2 h. The OD curve obtained at 0.5 h after the 4^th^ irradiation was the most linear (R^2^ = 0.992).

### Sensitivity of the OD curves

The values of slopes of each OD curve, which indicate the dosimetric sensitivity of PRESAGE^REU^, are listed in Table 2 for both the RT and LT groups. The maximal sensitivities were observed for the OD curves obtained at 0.5 h after the initial irradiation, for both the RT and LT groups (0.090 ΔOD/Gy for the RT group and 0.082 ΔOD/Gy for the LT group). With increasing the reading time, the sensitivities of the OD curves generally decreased, for both the RT and LT groups.

**Table 2:** Sensitivity changes of PRESAGE^REU^.

**Reading time after irradiation (hours)**	1^st^ irradiation	2^nd^ irradiation	3^rd^ irradiation	4^th^ irradiation
	Room temperature			
0.5	0.090	0.046	0.029	0.027
2	0.080	0.012	0.027	0.032
4	0.064	-0.003	0.024	0.036
6	0.051	-0.004	0.024	0.034
12	0.026	-0.003	0.025	0.034
24	0.005	-0.006	0.026	0.037
48	-0.002	-0.002	0.026	0.022
84	< 0.001	-0.001	0.024	0.032
	Low temperature			
0.5	0.082	0.049	0.045	0.056
2	0.059	0.031	0.033	0.037
4	0.054	0.021	0.023	0.032
6	0.043	0.013	0.016	0.023
12	0.016	0.005	0.004	0.013
24	0.004	0.001	0.001	0.006
48	-0.004	-0.001	< 0.001	0.004
84	-0.007	0.001	-0.002	0.004

In the case of the RT group, the sensitivity decreased as the reading time increased, for measurements performed following the 1^st^ and 2^nd^ irradiations. However, the sensitivity did not change with the reading time for measurements performed following the 3^rd^ and 4^th^ irradiations. In these cases, the sensitivity was always low, remaining at nearly 33% of the maximal sensitivity (0.03 ΔOD/Gy). For the OD curves obtained following the 1^st^ irradiation, if the reading time was less than or equal to 6 h, the sensitivity was above 45% of the maximal sensitivity, *i*.*e*., higher than 0.045 ΔOD/Gy. For the OD curves obtained following the 2^nd^ irradiation, the sensitivity was below 0.013 ΔOD/Gy, except the sensitivity of the OD curve at the reading time of 0.5 h, which was 0.046 ΔOD/Gy.

In the case of the LT group, the sensitivity decreased as the reading time increased, regardless of the irradiation history. The sensitivities of the OD curves acquired at 0.5 h after irradiation were always above 50% of the maximal sensitivity, 0.041 ΔOD/Gy, regardless of the irradiation history. For the OD curves obtained after the 1^st^ irradiation, for the reading time less than or equal to 6 h, the sensitivity was above 50% of the maximal sensitivity, *i*.*e*., above 0.041 ΔOD/Gy.

### Deviations across individual PRESAGE^REU^ dosimeters irradiated by identical doses

When irradiating the PRESAGE^REU^ dosimeters, three cuvettes were irradiated at a time by the same dose. Percentage deviations of individual readings across these three PRESAGE^REU^ cuvettes, with respect to the average reading over these three PRESAGE^REU^ cuvettes are averaged over 1 Gy to 20 Gy for each reading time and listed in Table 3.

**Table 3:** Percent deviations of readings across PRESAGE^REU^ dosimeters at each reading time.

	Room temperature	Low temperature	Room temperature	Low temperature
Reading time after irradiation (hours)	1^st^ irradiation		2^nd^ irradiation	
0.5	4.5 ± 5.7	8.4 ± 5.8	4.2 ± 2.9	7.2 ± 5.9
2	14.1 ± 14.5	11.2 ± 9.0	18.7 ± 17.5	26.4 ± 19.3
4	15.1 ± 19.0	18.4 ± 18.5	19.8 ± 122.5	27.8 ± 25.5
6	15.0 ± 10.4	11.7 ± 9.2	27.1 ± 182.1	27.2 ± 20.6
12	326.9 ± 921.9	132.1 ± 152.5	23.6 ± 137.7	3.4 ± 190.3
24	80.1 ± 303.5	2.6 ± 158.9	72.0 ± 172.9	35.7 ± 102.0
48	303.2 ± 562.9	337.2 ± 491.1	148.2 ± 262.4	4.1 ± 19.9
84	195.3 ± 804.6	239.3 ± 598.9	15.4 ± 187.9	5.1 ± 49.0
	3^rd^ irradiation		4^th^ irradiation	
0.5	10.3 ± 6.5	5.3 ± 4.4	30.5 ± 33.1	5.7 ± 3.1
2	9.7 ± 5.7	16.3 ± 7.9	32.1 ± 24.2	9.1 ± 5.8
4	11.6 ± 5.6	10.4 ± 6.5	10.5 ± 8.6	6.8 ± 4.4
6	31.0 ± 23.4	14.1 ± 9.6	18.1 ± 16.0	12.7 ± 10.3
12	15.1 ± 7.8	129.8 ± 159.9	14.2 ± 88.2	12.0 ± 8.9
24	7.9 ± 7.5	384.2 ± 1032.6	20.4 ± 35.2	85.9 ± 150.1
48	13.0 ± 3.4	366.4 ± 836.7	44.7 ± 84.2	59.9 ± 51.4
84	18.6 ± 9.1	79.8 ± 44.3	11.7 ± 10.9	64.1 ± 75.5

For dosimeters in the RT group, the average percent deviations were under 5% at 0.5 h after the 1^st^ and 2^nd^ irradiations (4.5% ± 5.7% for the 1^st^ irradiation and 4.2% ± 2.9% for the 2^nd^ irradiation). For readings at 0.5 h after the 3^rd^ and 4^th^ irradiations, the average percent deviations were above 10%. In general, percent deviation increased with increasing the reading time after irradiation. For reading times larger than or equal to 2 h, percent deviation was in general above 10%.

For dosimeters in the LT group, the average percent deviations were under 6% at 0.5 h after the 3^rd^ and 4^th^ irradiations (5.3 ± 4.4% for the 3^rd^ irradiation and 5.7% ± 3.1% for the 4^th^ irradiation). For readings at 0.5 h after the 1^st^ and 2^nd^ irradiations, the average percent deviations were under 9% (8.4% ± 5.8% for the 1^st^ irradiation, 7.2 ± 5.9% for the 2^nd^ irradiation). For longer reading times after irradiation, percent deviation in general was above 10%.

### Dose rate dependence

**Fig 5:**
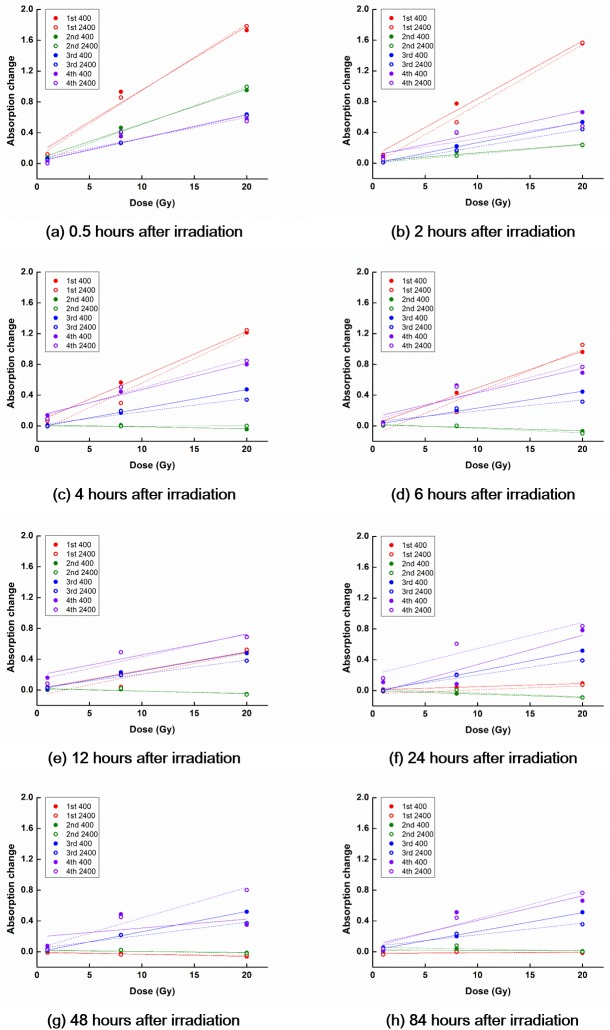
Dose-rate dependence of OD curves, for dosimeters at room temperature. The OD curves for dose rates of 400 MU/min and 2400 MU/min are shown with solid and dashed lines, respectively.

**Fig 6:**
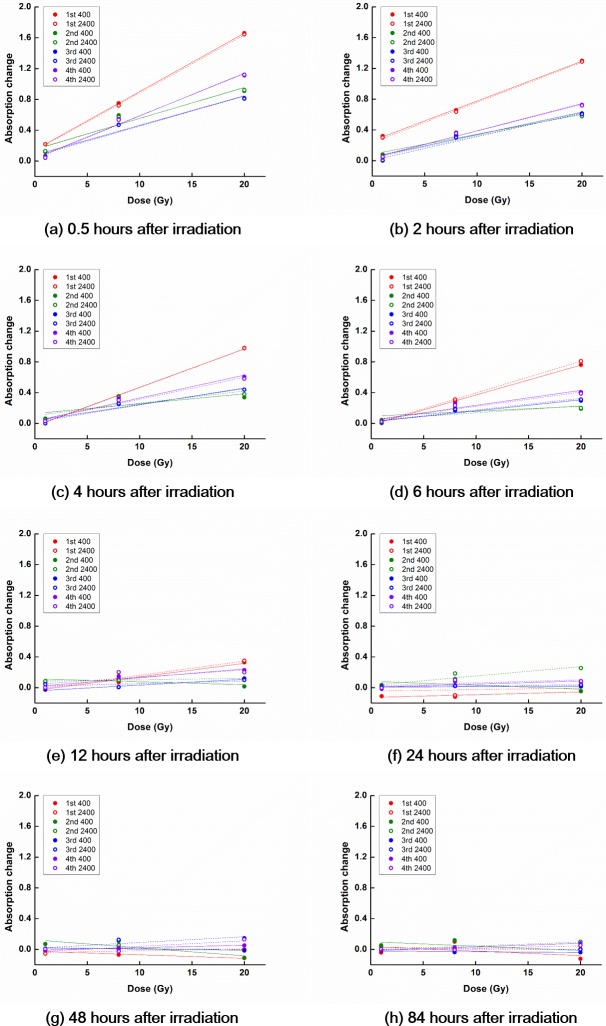
Dose-rate dependence of OD curves, for dosimeters at low temperature. The OD curves for dose rates of 400 MU/min and 2400 MU/min are shown with solid and dashed lines, respectively.

The OD curves for irradiation with the same dose but delivered at different dose rates (400 MU/min vs. 2400 MU/min) are shown in Fig 5 for dosimeters in the RT group. The corresponding OD curves for dosimeters in the LT group are shown in Fig 6.

In the case of the RT group, no considerable differences between the OD curves generated for irradiating at low and high dose rates were observed at 0.5 h after irradiation, regardless of the irradiation history. However, for measurements with longer reading times following irradiation, noticeable differences between OD curves were observed across low and high dose-rate irradiations.

In the case of the LT group, no considerable differences between the OD curves generated for irradiating at low and high dose rates were observed for measurements performed less than 6 h following irradiation, regardless of the irradiation history. Similar to the RT group, for measurements with longer reading times following irradiation, noticeable differences between OD curves were observed across low and high dose-rate irradiations.

### Dependence of OD curves on repeated usage of PRESAGE^REU^

**Fig 7:**
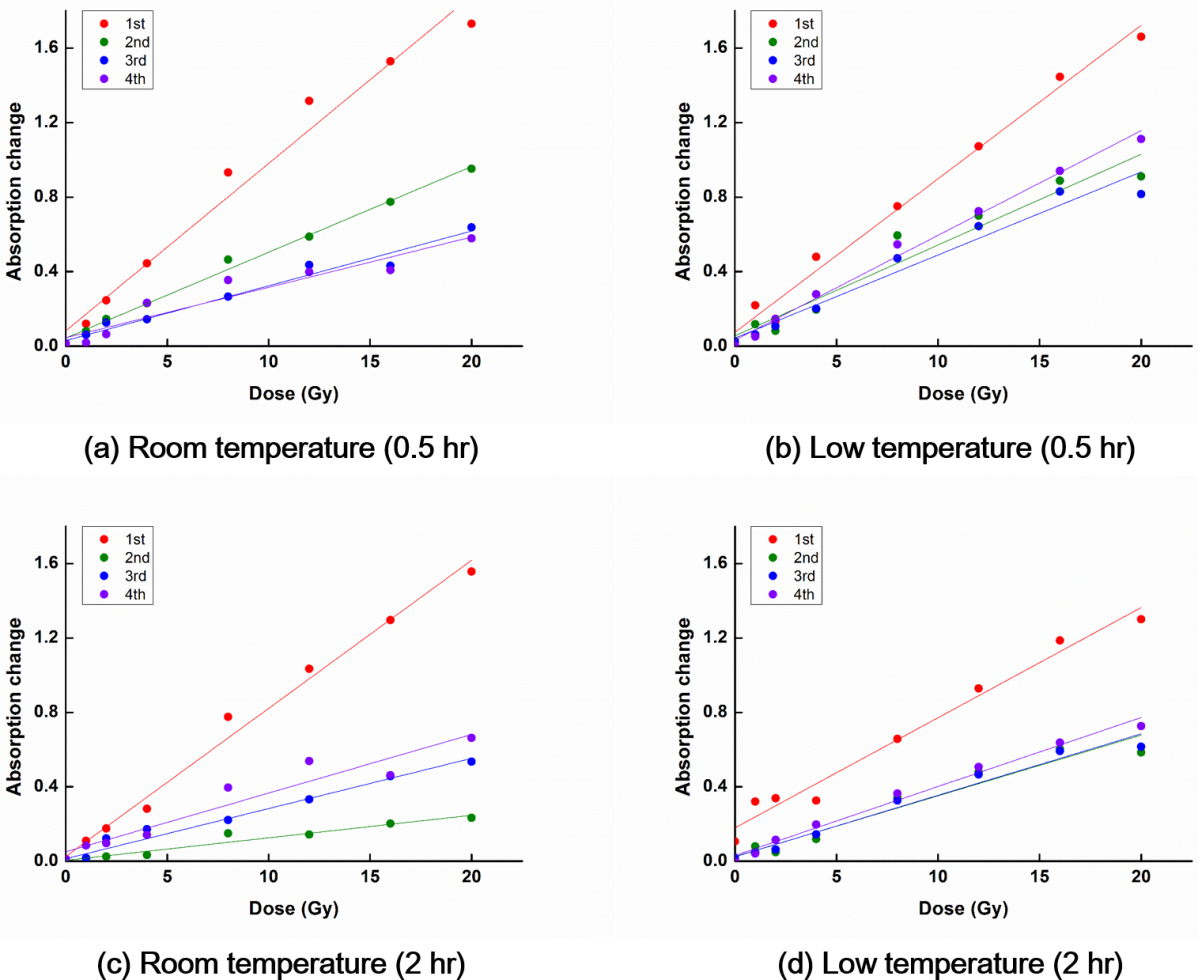
Changes in the OD curves incurred by repeated usage of PRESAGE^REU^, for measurements performed at 0.5 h and 2 h after irradiation. The sensitivity to the initial irradiation was higher than that to the repeat irradiation, for both the RT and LT groups.

The changes in the OD curves incurred by repeated usage, for measurements performed at 0.5 h and 2 h after irradiation are shown in Fig 7.

For the RT group, for the reading time of 0.5 h, the sensitivity of the OD curves decreased with increasing the number of irradiations. However, for the reading times of 2, 4, and 6 h, the sensitivity associated with the 1^st^ irradiation was the largest while that associated with the 2^nd^ irradiation was the smallest. The sensitivity associated with the 3^rd^ irradiation increased compared with that associated with the 2^nd^ irradiation, and the sensitivity associated with the 4^th^ irradiation increased compared with that associated with the 3^rd^ irradiation. For the same reading time, the OD curves differed, depending on the irradiation history.

For the LT group, for the reading time of 0.5 h, the sensitivity of the OD curves associated with the 1^st^ irradiation was the largest. The OD curve sensitivity associated with the 2^nd^ irradiation decreased compared with that associated with the 1^st^ irradiation, and the sensitivity associated with the 3^rd^ irradiation decreased compared with that associated with the 2^nd^ irradiation. However, the sensitivity associated with the 4^th^ irradiation increased compared with those associated with the 2^nd^ and 3^rd^ irradiations. For the reading times of 2, 4, and 6 h, the sensitivity associated with the 1^st^ irradiation was the largest while that associated with the 2^nd^ irradiation was the smallest, similar to the results for the RT group. The sensitivity associated with the 3^rd^ irradiation increased compared with that associated with the 2^nd^ irradiation; however, those were almost the same, differently from the results for the RT group. The sensitivity associated with the 4^th^ irradiation increased more, compared with that associated with the 3^rd^ irradiation. Qualitatively, the dependence of the OD curves on the number of irradiations was similar for the LT and RT groups. However, the differences between the OD curves associated with the 2^nd^ and 3^rd^ irradiations were much smaller for the LT group compared with the RT group.

## Discussion

In this study, we investigated dosimetric characteristics of the reusable 3D radiochromic dosimetry material, PRESAGE^REU^. We investigated the effects of the number of reuses, the measurement time post-irradiation, the temperature, and the dose rate, on the linearity and sensitivity associated with PRESAGE^REU^. For the RT group, the temporal decay of the dosimeter signal following the initial irradiation was regular; however, the decay patterns following re-irradiations were irregular. For the 2^nd^ irradiation of the RT group, PRESAGE^REU^ signals reached the background level only at 6 h following irradiation, while for the 1^st^ irradiation the signal reached the background level at 48 h following irradiation. The PRESAGE^REU^ signals in response to the 3^rd^ and 4^th^ irradiations of dosimeters in the RT group did not decay, and were not fully cleared out. On the contrary, for dosimeters in the LT group, the temporal decay following the 3^rd^ and 4^th^ irradiations was more stable than that in response to the 1^st^ and 2^nd^ irradiations. In the case of the LT group, PRESAGE^REU^ signals reached the background level at 48 h following irradiation, regardless of the irradiation history. Based on the decay patterns, we conclude that reusing PRESAGE^REU^ at room temperature does not yield reliable results. Considering both linearity and sensitivity of the OD curves, R^2^ above 0.95 and sensitivity above 0.04 ΔOD/Gy were observed following the 1^st^ irradiation (reading time ≤ 6 h) and 2^nd^ irradiation (reading time = 0.5 h) for the RT group, while those values were observed for the LT group following the 1^st^ irradiation (reading time ≤ 2 h), and the 3^rd^ and 4^th^ irradiations (both reading times = 0.5 h). In addition, considering the reproducibility of signals in response to the same dose, at the reading time 0.5 h following the 1^st^ and 2^nd^ irradiations dosimeters in the RT group yielded reliable values (average deviations among dosimeters < 5%), while for dosimeters in the LT group reliable values were obtained following the 3^rd^ and 4^th^ irradiations, for the same reading times of 0.5 h (average deviations among dosimeters < 6%). In these cases, the PRESAGE^REU^ dosimeters exhibited no dependence on the dose rate, as shown in Figs 5 and 6. Thus, it seems that at room temperature PRESAGE^REU^ dosimeters can be used up to two times. At lower temperatures (such as 10°C) PRESAGE^REU^ dosimeters can likely be reused. The relationship between the signals (ΔOD) and doses, *i*.*e*., the OD curves, should be re-defined for every reuse because the OD curves depend on the irradiation history of PRESAGE^REU^.

At room temperature, PRESAGE^REU^ signals decay with time since irradiation [14]. This was observed in most of the RT and LT cases in this study; however, it was not observed following the 3^rd^ and 4^th^ irradiations at room temperature, as shown in Fig 3. For the 3^rd^ and 4^th^ irradiations, the positive linear relationships between the values of ΔOD and doses were still observed because we subtracted the high remaining background values. Although we obtained OD curves with positive slopes, the results of the 3^rd^ and 4^th^ irradiations were not reliable, owing to the large deviation across dosimeters in response to the same dose, and owing to low R^2^ of the obtained OD curves. Therefore, at room temperature, the reuse of PRESAGE^REU^ seems limited, and in the present study, reliable responses were observed for up to two reuses.

For both the RT and LT groups, no noticeable differences were observed between the OD curves generated with the low dose rate (400 MU/min) and those generated with the high dose rate (2400 MU/min), when the reading time was 0.5 h. However, considerable differences between the low and high dose rate cases were observed as the reading time increased. Considering the large deviations across the PRESAGE^REU^ dosimeters for the reading times larger than 0.5 h (Table 3), the large differences between the PRESAGE^REU^ signals for low and high dose-rate irradiations and reading times larger than or equal to 2 h stemmed from the poor reproducibility of dosimeters at large reading times, rather than from the dose-rate differences. Because no dose-rate dependence of PRESAGE^REU^ was observed for either the RT or LT groups at the reading time of 0.5 h, it seems that PRESAGE^REU^ has no dose-rate dependence.

The results of the present study differ from those of the previous study by Pierquet *et al*. [14]. They showed reliable OD curves even for multiple re-irradiations at room temperature when signals were read immediately after irradiation, *i*.*e*., when the reading times were 0 h. Moreover, they showed, for repeated usage, that OD curves exhibit trends different to those observed by us here. In those previous studies, the sensitivity of PRESAGE^REU^ dosimeters increased after repeated usage, compared with the initial sensitivity. By contrast, in our study, the sensitivity of PRESAGE^REU^ dosimeters decreased after repeated usage, compared with the initial sensitivity. Pierquet *et al*. hypothesized that this increase in sensitivity on repeated usage was owing to the short time between the manufacturing of PRESAGE^REU^ and its 1^st^ irradiation. They claimed that the PRESAGE^REU^ dosimeters in their study may not have fully complete the post-manufacturing curing process before being irradiated for the first time. Such incomplete curing of PRESAGE^REU^ is not likely to affect the results of our study, because this study started two months after acquiring the PRESAGE^REU^ dosimeters. Moreover, it took additional time between manufacturing of the PRESAGE^REU^ used in this study and acquisition of the PRESAGE^REU^ since we purchased the PRESAGE^REU^ dosimeters from abroad. Instead of an increase in the sensitivity incurred by reusing PRESAGE^REU^, we observed a decrease and then an increase in the sensitivity with increasing the number of reuses, for both the RT and LT groups. The mechanism underlying this phenomenon will be investigated and reported elsewhere. On the other hand, the sensitivity of PRESAGE^REU^ in the previous study was ~0.04 ΔOD/Gy, similar to those in our study (0.046 ΔOD/Gy for the 2^nd^ irradiation of the RT group at the reading time of 0.5 h following irradiation and 0.05 ΔOD/Gy averaged over the results for 2^nd^ to 4^th^ irradiations of the LT group at the reading time of 0.5 h following irradiation) [14].

Since the ΔOD of PRESAGE^REU^ decreases with time after irradiation, the reading time after irradiation should be precise, for obtaining accurate dosimetry, especially when the reading time is short. A faster decay at short reading times compared with long reading times could increase the dosimetric errors to use PRESAGE^REU^ with short reading times. In this respect, a long reading time seems beneficial for a stable PRESAGE^REU^ dosimetry. However, the results of this study show that the deviations across PRESAGE^REU^ dosimeters, irradiated by the same dose, increase as the reading time increases. The decreased signals at these later times results in unreliable dosimetry. Therefore, the ΔOD values should be acquired as soon as possible after irradiation at a consistent reading time.

## Conclusions

The reusability of PRESAGE^REU^ at room temperature was not satisfactory, with PRESAGE^REU^ usability extending only to two irradiations. The behavior of PRESAGE^REU^ at low temperature (approximately 10°C) was different from that at room temperature, and it seems that at low temperature PRESAGE^REU^ stabilized with increasing the number of reuses. Although PRESAGE^REU^ reusability at low temperatures and short reading times post-irradiation seems feasible, the OD curve should be defined for every reuse because of its dependence on the number of reuses. In addition, for reliable PRESAGE^REU^ dosimetry, the ΔOD values should be read at short reading times and the reading times must be consistent. Accurate PRESAGE^REU^–based dosimetry should be performed with care.

## Supporting information

S1 Table This is the pre-irradiation optical density (OD) data. (DOCX)
