# Supplementary material for: Dosimetric characteristics of a reusable 3D radiochromic dosimetry material
Source: PLoS One. 2017 Jul 13;12(7):e0180970. doi: 10.1371/journal.pone.0180970 (PMC5509250; doi:10.1371/journal.pone.0180970)
Supplement: S1 Table — (DOCX) [file pone.0180970.s001.docx]

**S1 Table. Pre-irradiation optical density (OD) data.**

| Reading time after irradiation (hours) | 1^st^ irradiation | 2^nd^ irradiation | 3^rd^ irradiation | 4^th^ irradiation |
| --- | --- | --- | --- | --- |
|  | Room temperature | | | |
| 0.5 | 0.002 ± 0.000 | 0.016 ± 0.001 | -0.011 ± 0.001 | 0.014 ± 0.001 |
| 2 | -0.008 ± 0.000 | 0.001 ± 0.000 | -0.050 ± 0.004 | 0.0123 ± 0.001 |
| 4 | 0.008 ± 0.000 | 0.011 ± 0.001 | -0.010 ± 0.001 | 0.061 ± 0.005 |
| 6 | 0.010 ± 0.001 | 0.005 ± 0.000 | 0.013 ± 0.001 | 0.016 ± 0.001 |
| 12 | 0.014 ± 0.001 | 0.011 ± 0.001 | -0.002 ± 0.000 | 0.206 ± 0.024 |
| 24 | -0.029 ± 0.002 | 0.067 ± 0.002 | -0.034 ± 0.004 | 0.111 ± 0.017 |
| 48 | -0.016 ± 0.001 | 0.019 ± 0.001 | 0.002 ± 0.000 | 0.145 ± 0.020 |
| 84 | -0.007 ± 0.000 | 0.007 ± 0.000 | 0.008 ± 0.000 | 0.217 ± 0.024 |
|  | Low temperature | | | |
| 0.5 | 0.027 ± 0.002 | 0.026 ± 0.002 | 0.021 ± 0.001 | 0.004 ± 0.000 |
| 2 | 0.106 ± 0.008 | 0.106 ± 0.009 | 0.019 ± 0.001 | -0.002 ± 0.000 |
| 4 | 0.046 ± 0.003 | 0.014 ± 0.001 | 0.001 ± 0.000 | -0.005 ± 0.000 |
| 6 | 0.053 ± 0.003 | 0.010 ± 0.001 | 0.023 ± 0.001 | -0.001 ± 0.000 |
| 12 | 0.242 ± 0.018 | 0.012 ± 0.001 | 0.000 ± 0.000 | -0.007 ± 0.000 |
| 24 | 0.099 ± 0.007 | 0.011 ± 0.001 | 0.006 ± 0.000 | 0.001 ± 0.000 |
| 48 | 0.005 ± 0.000 | 0.045 ± 0.002 | 0.016 ± 0.001 | -0.001 ± 0.000 |
| 84 | 0.078 ± 0.006 | 0.023 ± 0.001 | 0.041 ± 0.002 | -0.024 ± 0.001 |

**Note: the values of OD in this table = OD of a dosimeter for irradiation – OD of dosimeters in the control group, which did not irradiated but placed near the dosimeters for irradiation in order to quantify and eliminate the environmental effect**
